# Supplementary material for: Context-dependent function of TSLP and IL-1β in skin allergic sensitization and atopic march
Source: Nat Commun. 2022 Sep 1;13:4703. doi: 10.1038/s41467-022-32196-1 (PMC9437001; doi:10.1038/s41467-022-32196-1)
Supplement: Supplementary file 1 — Supplementary Information [file 41467_2022_32196_MOESM1_ESM.pdf]

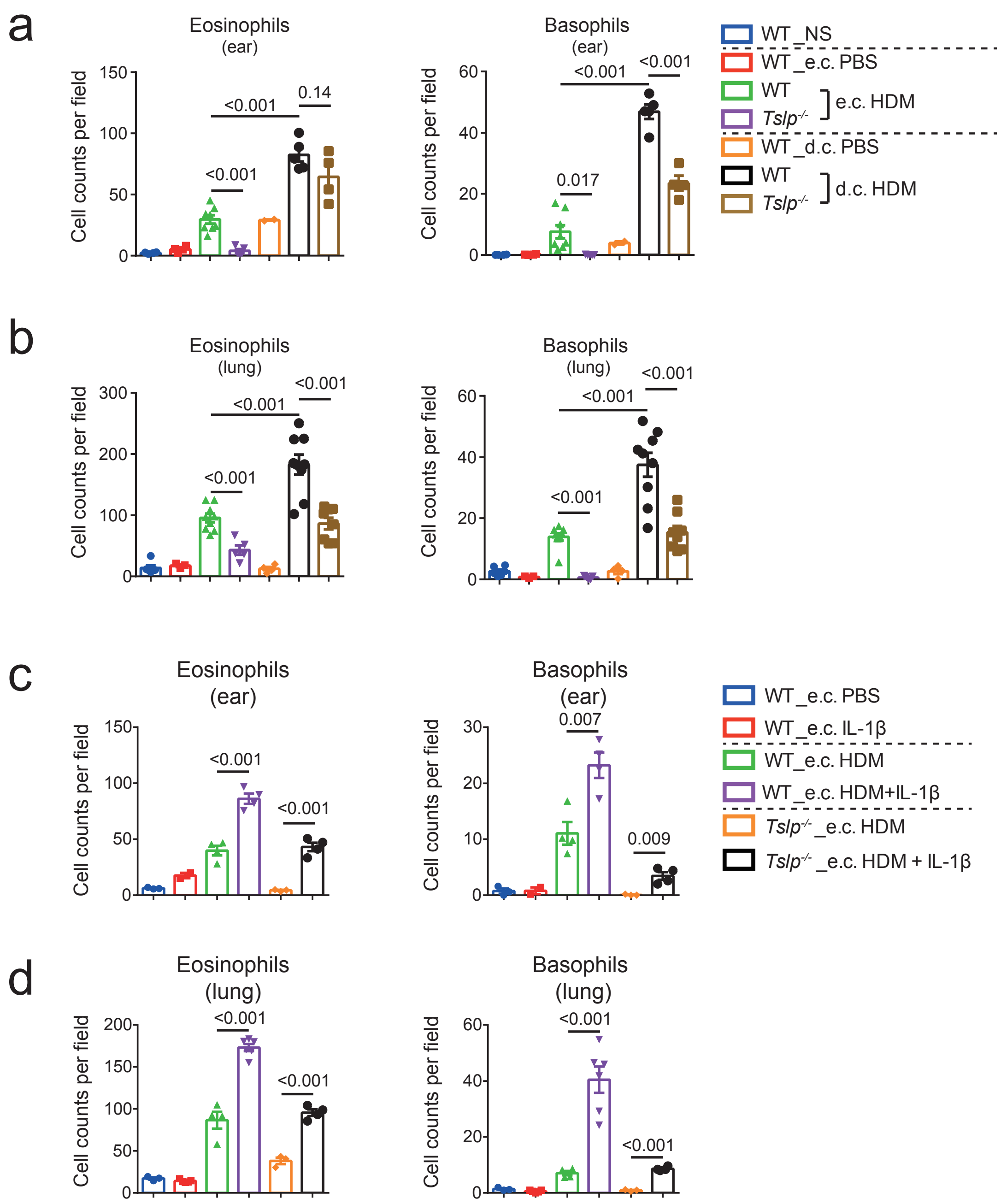

Supplementary Figure 1. Summary of cell counts for skin- or lung-infiltrating eosinophils and basophils from immunostained paraffin sections. One point corresponds to the average number of positively stained cells per microscopic field (at x 20 magnification) from 5 microscopic fields of the section of one mouse biopsy. **(a-b)** Mice were treated as described in **Fig. 1d**. Cell counts of eosinophils and basophils in ears (**a**; related to **Fig. 1e;** n=4, 4, 8, 5, 2, 5, 4 mice) or lungs (**b**; related to **Fig. 2d**; n=6, 3, 9, 5, 4, 9, 8 mice). (**c-d**) Mice were treated as described in **Fig. 3c**. Cell counts of eosinophils and basophils in ears (**c**; related to **Fig. 3e**; n=3, 2, 4, 4, 3, 4 mice) or lungs (**d**; related to **Fig. 4d**; n=3, 5, 4, 6, 3, 4 mice). Graphs show mean±SEM. Two-sided Student's t-test. p values are indicated. Source data are provided as a Source data file.

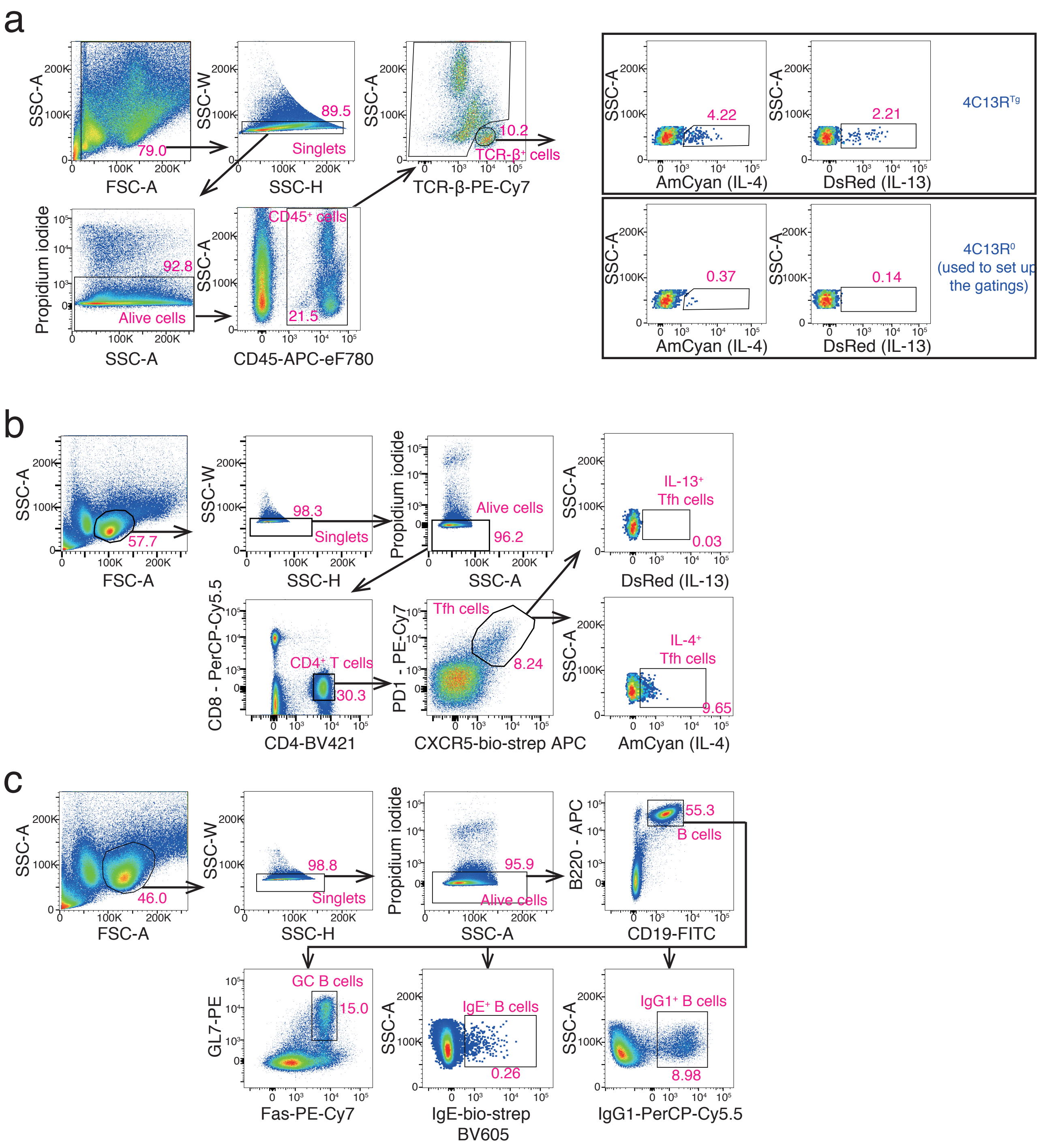

Supplementary Figure 2. Gating strategy for cells from e.c HDM- or d.c. HDM-treated 4C13R^{Tg} mice. a Gating strategy for IL-4 and IL-13 expression by TCRβ$^+$ cells in skin dermis, using Il4/Il13 dual reporter 4C13R^{Tg} mice. After excluding debris, doublets and dead cells, hematopoietic cells were gated as CD45$^+$. CD45$^+$TCR-β$^+$ cells were further gated for AmCyan (IL-4)$^+$ and DsRed(IL-13)$^+$ to examine cytokine production. Gates for Amcyan(IL-4) and DsRed(IL-13) are set with 4C13R^0 mice. **b** Gating strategy for Tfh cells in ear-draining lymph nodes (EDLNs). Alive single cells are gated for CD4 to identify CD4$^+$ T cells, Tfh cells are futher gated as CXCR5$^+$PD1$^+$ population. AmCyan (IL-4) or DsRed (IL-13) expressed in Tfh cells is gated in 4C13R^{Tg} mice, showing that Tfh cells express AmCyan (IL-4) but not DsRed (IL-13), as expected. **c** Gating strategy for B cells in EDLNs. B cells are gated as CD19$^+$B220$^+$ cells. Inside B cells, germinal center (GC) B cells are gated as GL7$^+$Fas$^+$ population. B cells are also gated for IgE$^+$ and IgG1$^+$ cells.

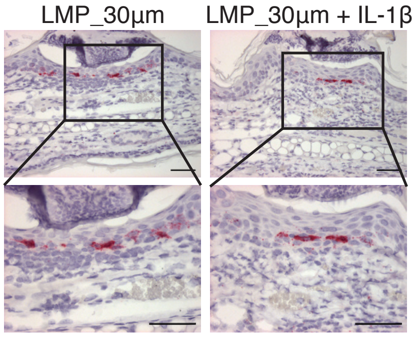

Supplementary Figure 3. Administration of IL-1β on LMP_30µm ears does not induce TSLP expression in ears. Ears from wildtype Balb/c mice were treated with LMP_30µm or LMP_30µm + 1µg of IL-1β (Cat No. 575106, Biolegend). Ears were collected 48h after treatment. RNAscope in situ hybridization for TSLP mRNA. Bar = 50µm for all pictures. Data are representative of 2 indep-dent experiments with similar results.

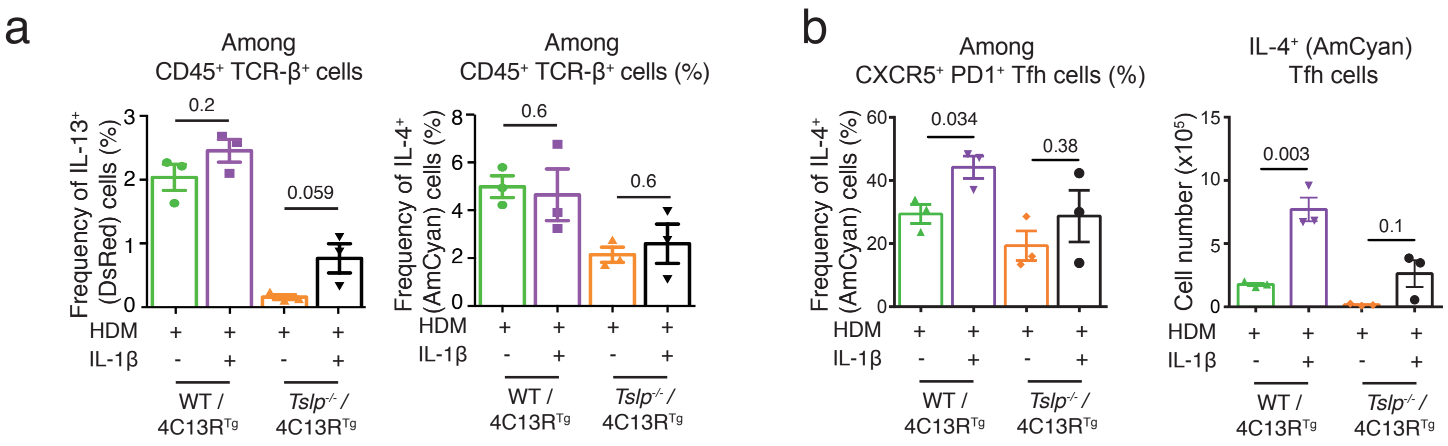

Supplementary Figure 4. Co-administration of IL-1β with e.c. HDM enhances IL-13 expression by Th2 cells in the skin and IL-4 expression by Tfh cells in ear-draining lymph nodes (EDLNs), in a TSLP-independent manner. Mice were treated with an experimental protocol as shown in **Fig. 3c**. **a** Frequency of IL-13^{+} (DsRed) or IL-4^{+} (AmCyan) cells among CD45^{+} TCR-β^{+} cells in ears, analyzed in WT and *Tslp*$^{-/-}$ mice in 4C13R^{Tg} background, showing that IL-1β administrated with e.c. HDM appears to enhance IL-13 expression by skin TCR-β^{+} cells in both WT and *Tslp*$^{-/-}$ mice. **b** Administration of IL-1β promotes IL-4-expressing Tfh cells in EDLNs. Graphs show mean±SEM; n=3 mice per group; Two-sided Student's t-test. p values are indicated. Data are representative of 2 independent experiments with similar results. Source data are provided as a Source data file.

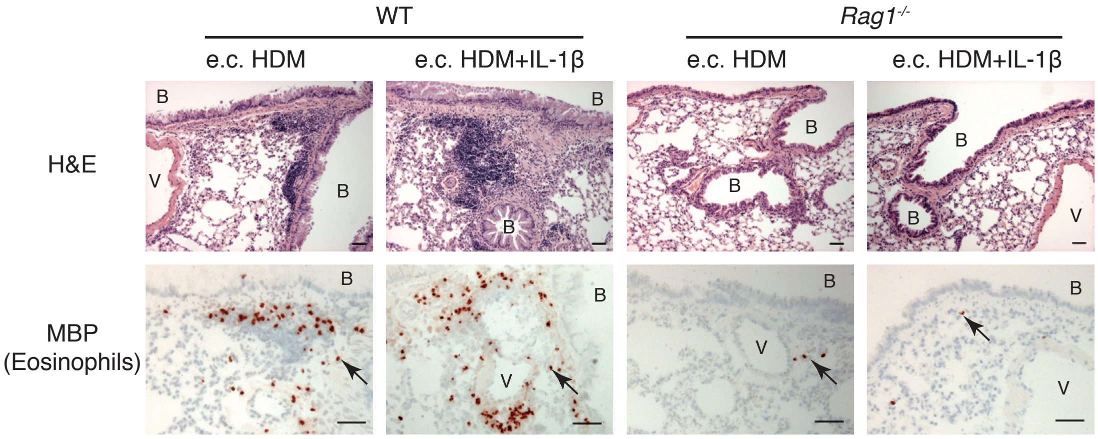

Supplementary Figure 5. Co-administration of IL-1β does not have any exacerbation of asthmatic phenotype in *Rag1$^{-/-}$* mice. Mice were treated as decribed in Fig. 3c. H&E and IHC for MBP (for eosinophils) in the lung sections from WT and *Rag1$^{-/-}$* mice. B: bronchiole; V: blood vessel. Bar = 50μm for all pictures. Data are representative of 2 independent experiments with similar results.

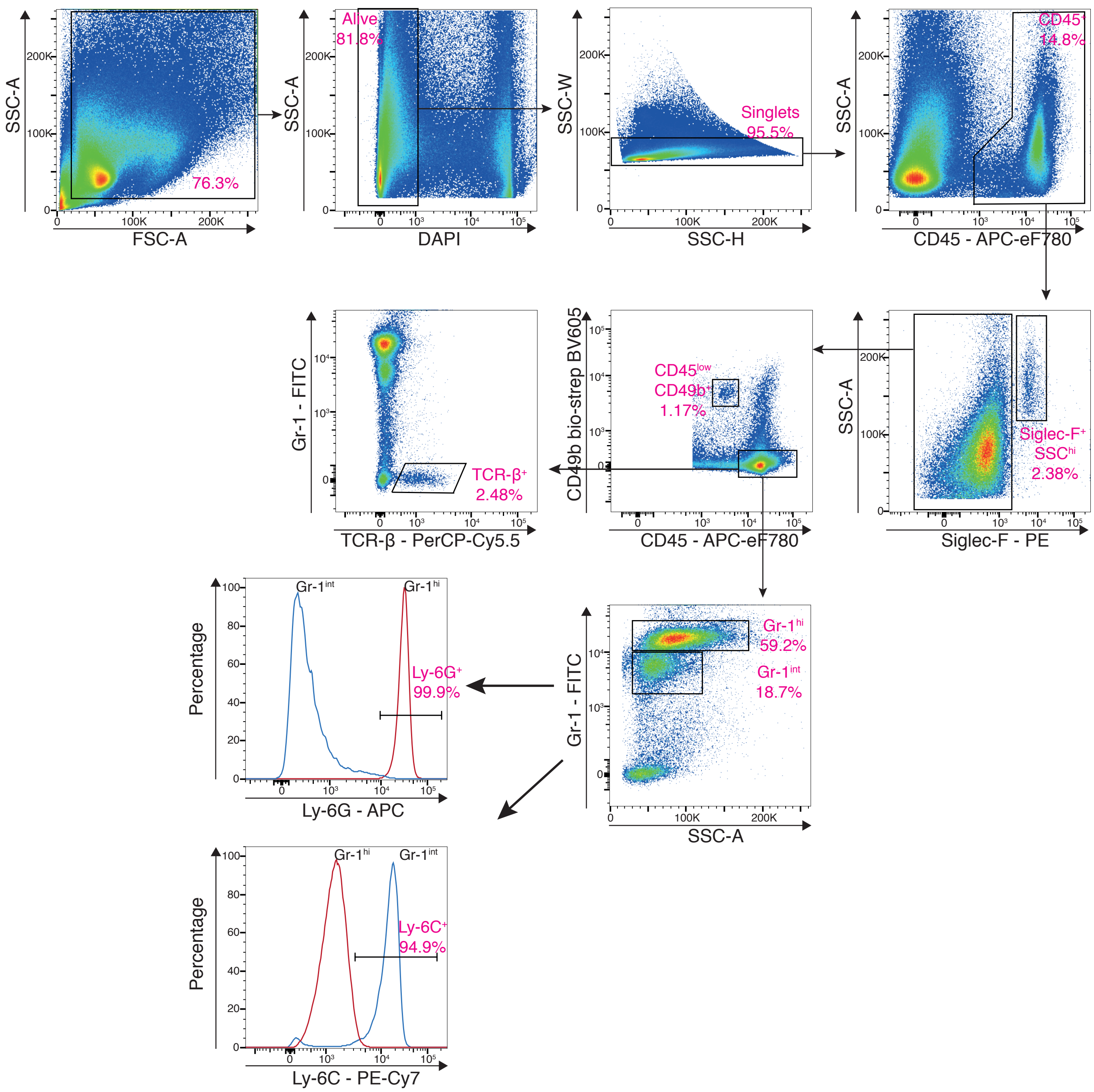

Supplementary Figure 6. Gating strategy for cells prepared from d.c. HDM-sensitized skin at 24h after treatment. After removing debris, dead cells and doublets, hematopoietic cells were gated as CD45^{+}. Eosinophils were gated as CD45^{+} Siglec-F^{+} SSC-A^{hi}. Basophils were gated as CD45^{low} Siglec-F^{-} CD49b^{+}. TCR-β^{+} T cells were gated as CD45^{+} Siglec-F^{-} CD49b^{-} TCR-β^{+}. Neutrophils were gated as CD45^{+} Siglec-F^{-} CD49b^{-} Gr-1^{hi}. Monocytes / macrophages were gated as CD45^{+} Siglec-F^{-} CD49b^{-} Gr-1^{int}. Gr-1^{hi} and Gr-1^{int} cells were further analysed for their expression of Ly-6G and Ly-6C, showing that as previously reported (Rose, S. et. al. 2012 PMID: 22213571), Gr-1^{hi} represent Ly-6G^{+} Ly-6C^{-} neutrophils, whereas Gr-1^{int} represent Ly6C^{+} Ly-6G^{-} monocytes/macrophages.

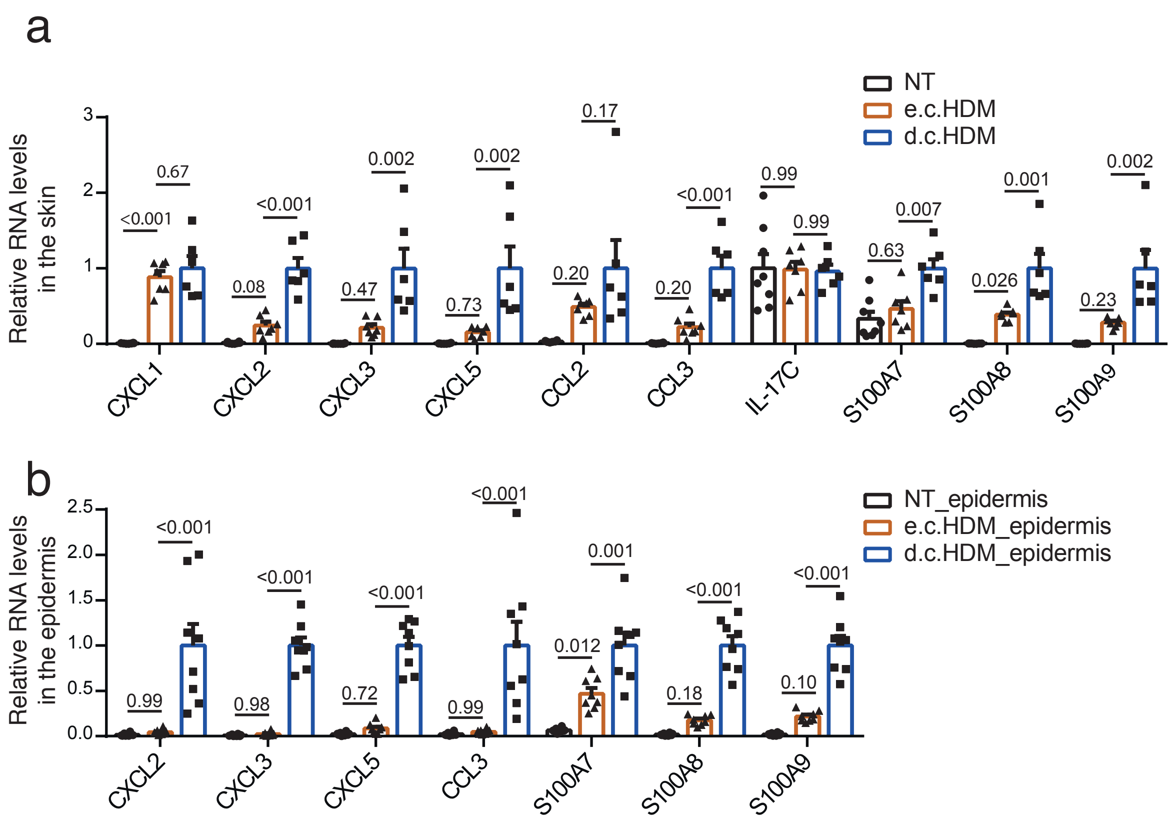

Supplementary Figure 7. Increased expression of neutrophil-attractant factors in d.c. HDM-treated skin. HDM (2µg) was applied on LMP_30*µ*m (e.c. HDM) and LMP_91*µ*m (d.c. HDM) ears of wildtype mice, and analysed 24 hrs later by quantitative RT-PCR (RT-qPCR). **a** Relative RNA levels of neutrophil chemoattractant factors in the skin from non-treated (NT), e.c. HDM and d.c. HDM-treated mice. n=6, 7 or 8 mice per group. **b** Relative RNA levels of neutrophil chemoattractant factors in the isolated epidermis (following the separation of epidermis and dermis from the ears incubated with 2.5 mg/ml dispase at 4°C overnight) from NT, e.c. HDM and d.c. HDM-treated mice. n=8 mice per group. Graphs show mean±SEM. One-way ANOVA test. p values are indicated. All data are representative of 2 independent experiments with similar results. Source data are provided as a Source data file.

Sequences of PCR primers are: CXCL1 (GCTGGGATTCACCTCAAGAA; AGGTGCCATCAGAGCAGTCT, 208 bp) ; CXCL2 (AGTGAACTGCGCTGTCAATG; TTCAGGGTCAAGGCAAACTT, 153 bp) ; CXCL3 (ATCCAGAGCTTGACGGTGAC; TCATCATGGTGAGGGGCTTC, 189 bp) ; CXCL5 (GTCCACAGTGCCCTACGG; ACTGGCCGTTCTTTCCACTG, 164 bp) ; CCL2 (GGTCCCTGTCATGCTTCTGG; CTTCTTGGGGTCAGCACAGA, 235 bp) ; CCL3 (GCAACCAAGTCTTCTCAGCG; TCTTTGGAGTCAGCGCAGAT, 181 bp) ; IL-17C (TGCTGGAAGCTGACACTCAC; CGTTGATGCATCCACGACAC, 123 bp) ; S100A7 (CTTGTCCCTGGAGGAGTTGA; GCTTGCCCAAGATGTACAGG, 167 bp) ; S100A8 (GGAAATCACCATGCCCTCTA; GAGATGCCACACCCACTTTT, 178 bp) ; S100A9 (AGATGGCCAACAAAGCACCT; TGTGTCCAGGTCCTCCATGA, 208 bp).

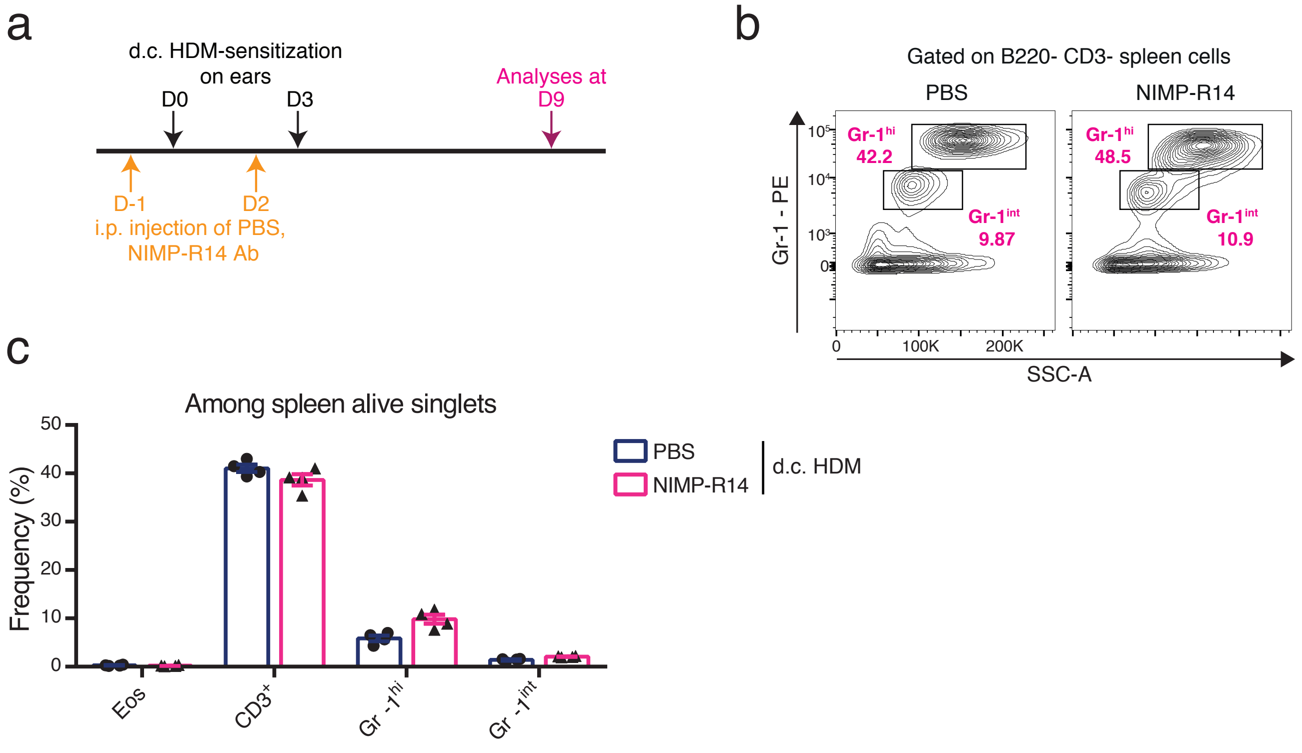

Supplementary Figure 8. Gr-1^{hi} and Gr-1^{int} cells are recovered at D9 following the administration of NIMP-R14 antibody. a Experimental protocol. Wild type Balb/c mice were intraperitoneally (i.p.) injected with PBS or NIMP-R14 antibody (100µg) at D-1 and D2. Mice were d.c. sensitized with HDM (2 µg) on LMP_91µm ears at D0 and D3. Spleen was analysed at D9 to examine the recovery of Gr-1^{hi} and Gr-1^{int} cells (the time point before intranasal (i.n.) HDM challenge; see the experimental protocol presented in Figure 6a). **b** Representative FACS plots of Gr-1^{hi} and Gr-1^{int} cells. **c** Comparison of frequency of eosinophils (eos), CD3^{+} cells, Gr-1^{hi} and Gr-1^{int} cells, showing similar Gr-1^{hi} and Gr-1^{int} cell percentages in NIMP-R14 and PBS-administrated mice. Data shown are mean±SEM; n=4 mice per group. Source data are provided as a Source data file.

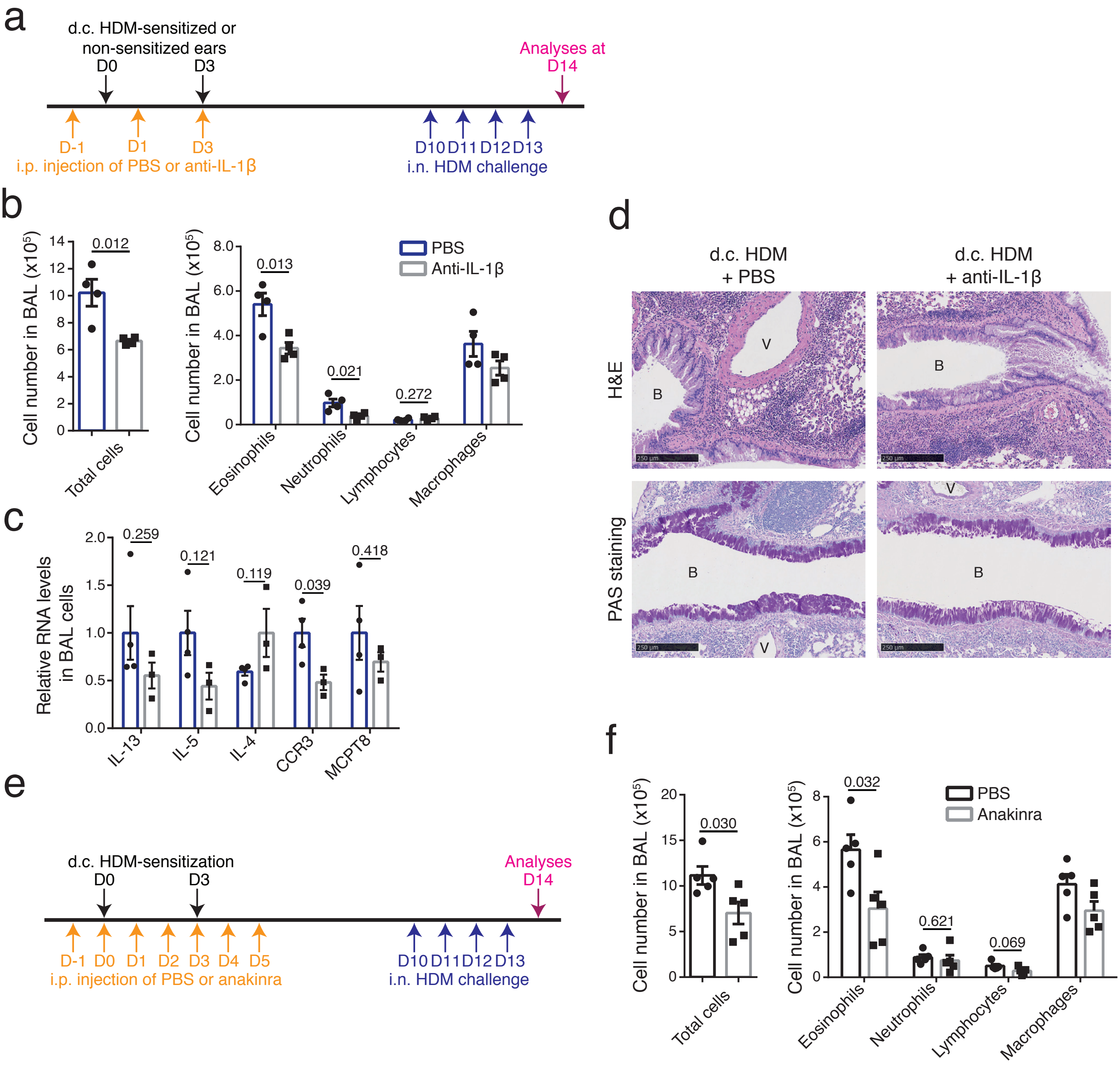

Supplementary Figure 9. The administration of anti-IL-1β antibody or Anakinra during d.c. HDM sensitization tends to reduce the subsequent asthmatic phenotypes. **a** Experimental protocol. Wild type Balb/c mice were intraperitoneally (i.p.) injected either with PBS or 200μg of anti-IL-1β antibody (clone B122, Cat No. BE0246, BioXCell) at D-1, D1 and D3. 2µg of HDM was applied on LMP_91 µm ears at D0 and D3 to induce d.c. HDM sensitization. Mice were then intranasally (i.n.) challenged with 2µg of HDM from D10 to D13. Mice were analysed at D14. **b** Total cell number and differential counting for eosinophils, neutrophils, lymphocytes and macrophages in BAL fluid. n=4 mice per group. **c** Relative RNA levels of genes in BAL cells. n=4, 3 mice per group. **d** Lung sections were stained with haematoxylin & eosin (H&E) for histological analyses or Periodic Acid Schiff (PAS) for goblet cell hyperplasia analyses. B: bronchiole. V: blood vessel. Bar=250 µm for all pictures. **e** Experimental protocol. Wild type Balb/c mice were i.p. injected either with PBS or 10 mg of anakinra (Kineret, Amgen) from D-1 to D5. 2µg of HDM was applied on LMP_91µm ears at D0 and D3 to induce d.c. HDM sensitization. Mice were then i.n. challenged with 2µg of HDM from D10 to D13, and analysed at D14. **f** Total cell number and differential counting for eosinophils, neutrophils, lymphocytes and macrophages in BAL fluid. n=5 mice per group. Graphs in **b**, **c, f** show mean±SEM; Two-sided Student's t-test. All data are representative of 2 independent experiments with similar results. Source data are provided as a Source data file.

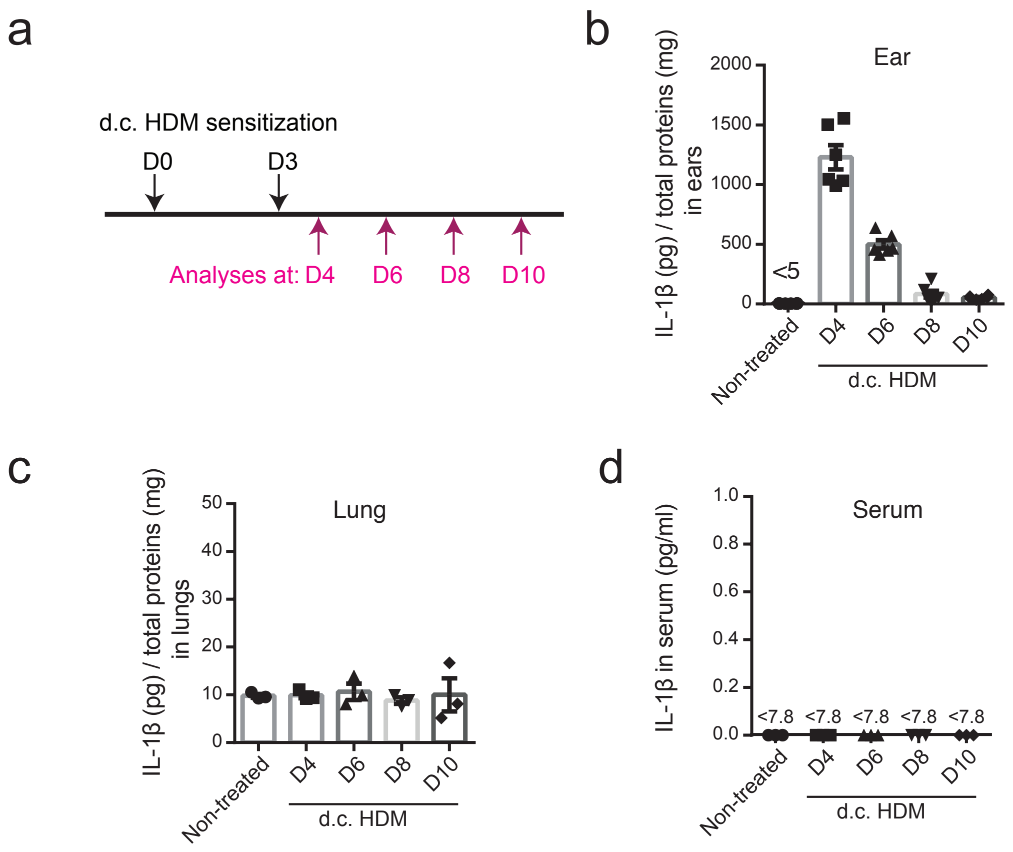

Supplementary Fig. 10. IL-1β levels in skin, lung and serum following d.c. HDM sensitization. a Experimental protocol. Wildtype Balb/c mice were dermacutaneously (d.c.) sensitized with HDM (2μg) at D0 and D3, and IL-1β levels in ears (**b**), lungs (**c**) and sera (**d**) were measured by ELISA at D4, D6, D8 or D10, showing that an increase in IL-1β was detected in the treated ears, which reduced with the time, but was not detected in the lung or in the serum. Data shown are mean ± SEM. n=6 mouse ears (**b**) and n=3 mice (**c, d**) per group. Source data are provided as a Source data file.
