# Supplementary material for: Context-dependent function of TSLP and IL-1β in skin allergic sensitization and atopic march
Source: Nat Commun. 2022 Sep 1;13:4703. doi: 10.1038/s41467-022-32196-1 (PMC9437001; doi:10.1038/s41467-022-32196-1)
Supplement: Supplementary file 3 — Reporting Summary [file 41467_2022_32196_MOESM3_ESM.pdf]

nature portfolio

Corresponding author(s): Mei LI

Last updated by author(s): May 7, 2022

Reporting Summary

Nature Portfolio wishes to improve the reproducibility of the work that we publish. This form provides structure for consistency and transparency in reporting. For further information on Nature Portfolio policies, see our Editorial Policies and the Editorial Policy Checklist.

Statistics

For all statistical analyses, confirm that the following items are present in the figure legend, table legend, main text, or Methods section.

n/a	Confirmed	
☐	☒	The exact sample size (n) for each experimental group/condition, given as a discrete number and unit of measurement
☐	☒	A statement on whether measurements were taken from distinct samples or whether the same sample was measured repeatedly
☐	☒	The statistical test(s) used AND whether they are one- or two-sided<br>*Only common tests should be described solely by name; describe more complex techniques in the Methods section.*
☐	☒	A description of all covariates tested
☐	☒	A description of any assumptions or corrections, such as tests of normality and adjustment for multiple comparisons
☐	☒	A full description of the statistical parameters including central tendency (e.g. means) or other basic estimates (e.g. regression coefficient) AND variation (e.g. standard deviation) or associated estimates of uncertainty (e.g. confidence intervals)
☐	☒	For null hypothesis testing, the test statistic (e.g. F, t, r) with confidence intervals, effect sizes, degrees of freedom and P value noted<br>*Give P values as exact values whenever suitable.*
☒	☐	For Bayesian analysis, information on the choice of priors and Markov chain Monte Carlo settings
☒	☐	For hierarchical and complex designs, identification of the appropriate level for tests and full reporting of outcomes
☒	☐	Estimates of effect sizes (e.g. Cohen's d, Pearson's r), indicating how they were calculated

Our web collection on statistics for biologists contains articles on many of the points above.

Software and code

Policy information about availability of computer code

Data collection	BD FACS Diva v8, NDP.scan
Data analysis	FlowJo v10 (BD Biosciences), GraphPad Prism 9, NDP.view 2

For manuscripts utilizing custom algorithms or software that are central to the research but not yet described in published literature, software must be made available to editors and reviewers. We strongly encourage code deposition in a community repository (e.g. GitHub). See the Nature Portfolio guidelines for submitting code & software for further information.

Data

Policy information about availability of data

All manuscripts must include a data availability statement. This statement should provide the following information, where applicable:

- Accession codes, unique identifiers, or web links for publicly available datasets
- A description of any restrictions on data availability
- For clinical datasets or third party data, please ensure that the statement adheres to our policy

The data supporting the findings are provided in the article, Supplementary information or Source data file. Source data are provided with this paper.

Field-specific reporting

Life sciences study design

All studies must disclose on these points even when the disclosure is negative.

Sample size	Sample size was determined according to our previous mouse studies and the recommendation from ethics committee of animal experimentation, and was based on the minimum required to achieve statistical significance. 3-6 mice per group were estimated sufficient to detect differences between groups based on the reproducibility between independent experiments.
Data exclusions	No data were excluded from the analysis.
Replication	The numbers of replication of the experiments are indicated in figure legends.
Randomization	Age- and sex-matched mice were randomly assigned to each group.
Blinding	For airway resistance analysis, researchers performing the analysis were blinded. The rest of the experiments were not performed in a blind manner as the researcher was aware of mouse genotype to establish the experimental cohorts, to do treatments, to follow the phenotype, collecting, and to analyze data. As it is mandatory to identify (a number is attributed to each mouse) and follow the phenotype of each individual mouse, this prevented blinding.

Reporting for specific materials, systems and methods

We require information from authors about some types of materials, experimental systems and methods used in many studies. Here, indicate whether each material, system or method listed is relevant to your study. If you are not sure if a list item applies to your research, read the appropriate section before selecting a response.

Materials & experimental systems

n/a	Involved in the study	
☐	☒	Antibodies
☒	☐	Eukaryotic cell lines
☒	☐	Palaeontology and archaeology
☐	☒	Animals and other organisms
☒	☐	Human research participants
☒	☐	Clinical data
☒	☐	Dual use research of concern

Methods

n/a	Involved in the study	
☒	☐	ChIP-seq
☐	☒	Flow cytometry
☒	☐	MRI-based neuroimaging

Antibodies

Antibodies used

For ELISA analyses, anti-TSLP (DY555) and anti-IL-1b (DY401) were from DuoSet ELISA kit from R&D Systems. Biotinylated anti-IgE (1:250, Catalog number: 553419, clone R35-118) and anti-IgG1 (1:250, Catalog number: 553441, clone A85-1) were from BD Biosciences.

For immunohistochemistry, anti-MBP (1:2000) was from Dr. James J. Lee (Mayo Clinic, Rochester).
Anti-MCPT8 (1:500, Catalog number: 647402, clone TUG8) was from Biolegend.
Biotinylated anti-IgG was from Vector Laboratories (1:300, Catalog number: PK-6104)

For flow cytometry:
anti-CD16/CD32 antibody (0.5:25, clone 93, eBioscience), Catalog number: 14-0161-85
anti-CD45 APC-eFluor780 (0.06:25, clone 30-F11), eBioscience, Catalog number: 47-0451-82
anti-CD45R/B220 APC (1.2:25, clone RA3-6B2), eBioscience, Catalog number: 17-0452-83
anti-GL7 PE (1.25:25, clone GL-7), eBioscience, Catalog number: 12-5902-82
anti-Gr-1 PE (Ly-6G/Ly-6C) (0.02:25, clone RB6-8C5), eBioscience, Catalog number: 12-5931-83
anti-TCR-β PerCP-Cy5.5 (1:25, clone H57-597), eBioscience, Catalog number: 45-5961-82
anti-CD3 FITC (1:25, clone 145-2C11), eBioscience, Catalog number:11-0031-82
anti-Ly-6C PE-Cy7 (0.3:25, clone HK1.4), eBioscience, Catalog number: 25-5932-82
anti-Ly-6G APC (1:25, clone 1A8-Ly6g), eBioscience, Catalog number: 17-9668-82
anti-CD49b biotin (0.5:25, clone DX5), eBioscience, Catalog number: 13-5971-82
anti-CD8a PerCP-Cy5.5 (0.5:25, clone 53-6.7), eBioscience, Catalog number: 45-0081-82
anti-CD19 PerCP-Cy5.5 (0.1:25, clone 1D3), eBioscience, Catalog number: 45-0193-82
anti-Gr-1 FITC (Ly-6G/Ly-6C) (0.05:25, clone RB6-8C5), BD Biosciences, Catalog number: 553127
anti-Siglec-F PE (0.5:25, clone E50-2440), BD Biosciences, Catalog number: 552126
anti-CD95 PE-Cy7 (1:25, clone Jo2), BD Biosciences, Catalog number: 557653
anti-CD19 FITC (1:25, clone 1D3), BD Biosciences, Catalog number: 553785
anti-CXCR5 biotin (1.5:25, clone 2G8), BD Biosciences, Catalog number: 551960

anti-IgE biotin (0.5:25, clone R35-72), BD Biosciences, Catalog number: 553414
anti-TCR-β PE-Cy7(0.5:25, clone H57-597), Biolegend, Catalog number: 109221
anti-CD4 BV421 (0.5:25, clone GK1.5), Biolegend, Catalog number: 100438
anti-PD-1 PE-Cy7 (2:25, clone RMP1-30), Biolegend, Catalog number: 109110
anti-IgG1 PerCP-Cy5.5 (1:25, clone RMG1-1), Biolegend, Catalog number: 406612
anti-CD45R/B220 PE-Cy7 (1.2:25, clone RA3-6B2), Biolegend, Catalog number: 103222
anti-IL-1b PE (2:100, clone 166931) R&D Systems, Catalog number: IC4013P

For in vivo administration, anti-Ly6G (clone 1A8, Catalog number: BP0075-1) and anti-IL-1b (clone B122, Catalog number: BE0246) were from BioXCell. Anti-GR1 clone NIMP-R14 was provided by S. F. Martin (University of Freiburg).

Validation

For flow cytometry:
anti-CD16/CD32 antibody (0.5:25, clone 93, eBioscience), Catalog number: 14-0161-85
https://www.thermofisher.com/antibody/product/CD16-CD32-Antibody-clone-93-Monoclonal/14-0161-82
Staining of BALB/c splenocytes with Anti-Mouse CD16/32 Purified followed by Anti-Rat IgG FITC

anti-CD45 APC-eFluor780 (0.06:25, clone 30-F11), eBioscience, Catalog number: 47-0451-82
https://www.thermofisher.com/antibody/product/CD45-Antibody-clone-30-F11-Monoclonal/47-0451-82
Staining of C57BL/6 bone marrow cells with Anti-Mouse CD45 APC-eFluor 780

anti-CD45R/B220 APC (1.2:25, clone RA3-6B2), eBioscience, Catalog number: 17-0452-83
https://www.thermofisher.com/antibody/product/CD45R-B220-Antibody-clone-RA3-6B2-Monoclonal/17-0452-82
Staining of C57BL/6 splenocytes with Anti-Human/Mouse CD45R (B220) APC

anti-GL7 PE (1.25:25, clone GL-7), eBioscience, Catalog number: 12-5902-82
https://www.thermofisher.com/antibody/product/GL7-Antibody-clone-GL-7-GL7-Monoclonal/12-5902-82
Staining of Con A-stimulated C57Bl/6 splenocytes with Anti-Mouse CD3 PerCP-eFluor® 710 and Anti-Human/Mouse GL7 PE.

anti-Gr-1 PE (Ly-6G/Ly-6C) (0.02:25, clone RB6-8C5), eBioscience, Catalog number: 12-5931-83
https://www.thermofisher.com/antibody/product/Ly-6G-Ly-6C-Antibody-clone-RB6-8C5-Monoclonal/12-5931-82
Total BALB/c bone marrow cell suspension was stained with Anti-Mouse Ly-6G (Gr-1) PE.

anti-TCR-β PerCP-Cy5.5 (1:25, clone H57-597), eBioscience, Catalog number: 45-5961-82
https://www.thermofisher.com/antibody/product/TCR-beta-Antibody-clone-H57-597-Monoclonal/45-5961-82
Staining of C57BL/6 splenocytes with Anti-Human/Mouse CD45R (B220) FITC and Anti-Mouse TCR beta PerCP-Cyanine5-5.

anti-CD3 FITC (1:25, clone 145-2C11), eBioscience, Catalog number:11-0031-82
https://www.thermofisher.com/antibody/product/CD3e-Antibody-clone-145-2C11-Monoclonal/11-0031-82
Staining of C57Bl/6 splenocytes with Anti-Mouse CD19 PE and Anti-Mouse CD3e FITC

anti-Ly-6C PE-Cy7 (0.3:25, clone HK1.4), eBioscience, Catalog number: 25-5932-82
https://www.thermofisher.com/antibody/product/Ly-6C-Antibody-clone-HK1-4-Monoclonal/25-5932-82
Staining of C57Bl/6 splenocytes with Anti-Mouse CD8a APC and Anti-Mouse Ly-6C PE-Cyanine7.

anti-Ly-6G APC (1:25, clone 1A8-Ly6g), eBioscience, Catalog number: 17-9668-82
hhttps://www.thermofisher.com/antibody/product/Ly-6G-Antibody-clone-1A8-Ly6g-Monoclonal/17-9668-82
Staining of C57Bl/6 bone marrow cells with Anti-Human/Mouse CD45R (B220) FITC and Anti-Mouse Ly-6G APC.

anti-CD49b biotin (0.5:25, clone DX5), eBioscience, Catalog number: 13-5971-82
https://www.thermofisher.com/antibody/product/CD49b-Integrin-alpha-2-Antibody-clone-DX5-Monoclonal/13-5971-82
Staining of BALB/c splenocytes with Anti-Mouse CD49b (Integrin alpha 2) Biotin followed by Streptavisin PE.

anti-CD8a PerCP-Cy5.5 (0.5:25, clone 53-6.7), eBioscience, Catalog number: 45-0081-82
https://www.thermofisher.com/antibody/product/CD8a-Antibody-clone-53-6-7-Monoclonal/45-0081-82
Staining of C57BL/6 splenocytes with Anti-Mouse CD8a PerCP-Cyanine5-5.

anti-CD19 PerCP-Cy5.5 (0.1:25, clone 1D3), eBioscience, Catalog number: 45-0193-82
https://www.thermofisher.com/antibody/product/CD19-Antibody-clone-eBio1D3-1D3-Monoclonal/45-0193-82
Staining of C57BL/6 splenocytes with Anti-Mouse CD3e FITC and Anti-Mouse CD19 PerCP-Cyanine5-5.

anti-Gr-1 FITC (Ly-6G/Ly-6C) (0.05:25, clone RB6-8C5), BD Biosciences, Catalog number: 553127
https://www.bdbiosciences.com/en-tw/products/reagents/flow-cytometry-reagents/research-reagents/single-color-antibodies-ruo/fitc-rat-anti-mouse-ly-6g-and-ly-6c.553127
This antibody is routinely tested by flow cytometric analysis. Other applications were tested at BD Biosciences Pharmingen during antibody development only.

anti-Siglec-F AlexaFluor 647 (0.5:25, clone E50-2440), BD Biosciences, Catalog number: 562680
https://www.bdbiosciences.com/en-nz/products/reagents/flow-cytometry-reagents/research-reagents/single-color-antibodies-ruo/alexa-fluor-647-rat-anti-mouse-siglec-f.562680

BALB/c bone-marrow cells were stained with FITC rat anti-mouse CD11b antibody and Alexa Fluor 647 Rat anti-mouse Sigle-F anitbody in the presence of purified Rat anti-mouse CD16/CD32 antibody.

anti-Siglec-F PE (0.5:25, clone E50-2440), BD Biosciences, Catalog number: 552126
https://www.bdbiosciences.com/en-nz/products/reagents/flow-cytometry-reagents/research-reagents/single-color-antibodies-ruo/pe-rat-anti-mouse-siglec-f.552126
BALB/c bone-marrow leukocytes were simultaneously stained with FITC-conjugated anti-mouse CD11b (Integrin αM chain) mAb M1/70 and PE-conjugated mAb E50-2440 in the presence of Mouse Fc Block.

anti-CD19 FITC (1:25, clone 1D3), BD Biosciences, Catalog number: 553785
https://www.bdbiosciences.com/en-nz/products/reagents/flow-cytometry-reagents/research-reagents/single-color-antibodies-ruo/fitc-rat-anti-mouse-cd19.553785
BABL/c splenocytes were stained with PE-conjugated anti-mouse CD3e mAb 145-2C11 in presence of FITC-conjugated mAb 1D3

anti-CXCR5 biotin (1.5:25, clone 2G8), BD Biosciences, Catalog number: 551960
https://www.bdbiosciences.com/en-nz/products/reagents/flow-cytometry-reagents/research-reagents/single-color-antibodies-ruo/biotin-rat-anti-mouse-cd185-cxcr5.551960
Splenocytes were stained with FITC Rat Anti-Mouse CD45R/B220 and Biotin Rat Anti-Mouse CD185 (CXCR5), followed by PE Streptavidin

anti-IgE biotin (0.5:25, clone R35-72), BD Biosciences, Catalog number: 553414
https://www.bdbiosciences.com/en-us/products/reagents/immunoassay-reagents/elisa/biotin-rat-anti-mouse-ige.553414

anti-TCR-β PE-Cy7 (0.5:25, clone H57-597), Biolegend, Catalog number: 109221
https://www.biolegend.com/fr-fr/products/pe-cyanine7-anti-mouse-tcr-beta-chain-antibody-4144
C57BL/6 mouse splenocytes stained with H57-597 PE/Cyanine7

anti-CD4 BV421 (0.5:25, clone GK1.5), Biolegend, Catalog number: 100438
https://www.biolegend.com/en-us/products/brilliant-violet-421-anti-mouse-cd4-antibody-7142
C57BL/6 mouse splenocytes were stained with CD3ε FITC and CD4 (clone GK1.5) Brilliant Violet 421

anti-PD-1 PE-Cy7 (2:25, clone RMP1-30), Biolegend, Catalog number: 109110
https://www.biolegend.com/fr-ch/products/pe-cyanine7-anti-mouse-cd279-pd-1-antibody-3612
Con A-stimulated (day-3) Balb/c mouse splenocytes stained with RMP1-30 PE/Cyanine7

anti-IgG1 PerCP-Cy5.5 (1:25, clone RMG1-1), Biolegend, Catalog number: 406612
https://www.biolegend.com/en-us/search-results/percp-cyanine5-5-anti-mouse-igg1-8488?GroupID=BLG3729
Human peripheral blood lymphocytes were stained with purified anti-human CD3 (clone UCHT1), followed by anti-mouse IgG1 (clone RMG1-1) PerCP/Cyanine5.5.

anti-CD45R/B220 PerCP-Cy5.5 (0.3:25, clone RA3-6B2), Biolegend, Catalog number: 103236
https://www.biolegend.com/de-at/products/percp-cyanine5-5-anti-mouse-human-cd45r-b220-antibody-4267
C57BL/6 mouse splenocytes stained with CD3 APC and CD45R/B220 (clone RA3-6B2) PerCP/Cyanine5.5

anti-CD45R/B220 PE-Cy7 (1.2:25, clone RA3-6B2), Biolegend, Catalog number: 103222
https://www.biolegend.com/de-de/products/pe-cyanine7-anti-mouse-human-cd45r-b220-antibody-1930
C57BL/6 mouse splenocytes stained with RA3-6B2 PE/Cyanine7

anti-IL-1b PE (2:100, clone 166931) R&D Systems, Catalog number: IC4013P
https://www.rndsystems.com/products/mouse-il-1beta-il-1f2-pe-conjugated-antibody-166931_ic4013p
Mouse splenocytes treated with LPS for 48 hours were stained with Rat Anti-Mouse IL-1 beta /IL-1F2 PE-conjugated Monoclonal Antibody. To facilitate intracellular staining, cells were fixed with Flow Cytometry Fixation Buffer and permeabilized with Flow Cytometry Permeabilization/Wash Buffer I.

For in vivo administration, anti-Ly6G (clone 1A8, Catalog number: BP0075-1) and anti-IL-1b (clone B122, Catalog number: BE0246) were from BioXCell.
https://bxcell.com/product/invivoplus-anti-m-ly-6g-2/
The 1A8 monoclonal antibody reacts with mouse Ly6G. Applications: in vivo neutrophil depletion
https://bxcell.com/product/m-il-1-beta/
The B122 monoclonal antibody reacts with precursor and mature secreted forms of mouse and rat IL-1β. Application: in vivo IL-1b neutralization

Anti-GR1 was given by S. F. Martin (University of Freiburg) and produced in house (clone NIMP-R14). Validation of neutrophil and monocytes / macrophage depletion is provided in the paper.

For ELISA analyses, anti-TSLP (DY555) and anti-IL-1b (DY401) were from DuoSet ELISA kit from R&D Systems.
https://www.rndsystems.com/products/mouse-tslp-duoset-elisa_dy555
https://www.rndsystems.com/products/mouse-il-1-beta-il-1f2-duoset-elisa_dy401

For immunohistochemistry:

Anti-MCPT8 (1:500, Catalog number: 647402, clone TUG8) was from Biolegend.
https://www.biolegend.com/en-us/products/purified-anti-mmcp-8-antibody-6266
Cell extracts from untransfected NIH/3T3 cells or NIH/3T3 cells transfected with a plasmid encoding mMCP-8-Flag tagged protein, using anti-mMCP-8, clone TUG8.

anti-MBP (1:2000) was from Dr. James J. Lee (Mayo Clinic, Rochester).
DOI: https://doi.org/10.4049/jimmunol.165.10.5509

Animals and other organisms

Policy information about studies involving animals; ARRIVE guidelines recommended for reporting animal research

Laboratory animals	4C13R dual reporter transgenic (Tg) mice were provided by Dr. W.E. Paul (NIH, USA). Tslp-/- mice were generated by Dr. Mei Li (IGBMC, France). Tslp-/- 4C13RTg compound mice were obtained by breeding 4C13R Tg and Tslp-/- mice. Balb/c and Rag1-/- mice were from the Jackson Laboratory. All mice were backcrossed to Balb/c background. All experimental mice were females, between 10 to 15 weeks old.<br>All mice were housed at a temperature of 22°C, humidity of 40-60% in a 12h light/12h dark cycle, with unlimited access to food and water.
Wild animals	No wild animals were used in this study.
Field-collected samples	No field-collected samples were used in this study.
Ethics oversight	Breeding and maintenance were performed under institutional guidelines, and all of the animal experiments were approved by the animal care and ethics committee of animal experimentation of the IGBMC, and by the Ministère de l'enseignement supérieur, de la recherche et de l'innovation.

Note that full information on the approval of the study protocol must also be provided in the manuscript.

Flow Cytometry

Plots

Confirm that:

- [x] The axis labels state the marker and fluorochrome used (e.g. CD4-FITC).
- [x] The axis scales are clearly visible. Include numbers along axes only for bottom left plot of group (a 'group' is an analysis of identical markers).
- [x] All plots are contour plots with outliers or pseudocolor plots.
- [x] A numerical value for number of cells or percentage (with statistics) is provided.

Methodology

Sample preparation	For preparation of dermal cells, ears were split into ventral and dorsal halves and incubated 1h at 37°C with 4 mg/ml Dispase (Gibco). Dermis was separated from epidermis and incubated 1h at 37°C with 1mg/ml collagenase D (Roche), 0.25 mg/ml DNase I (Sigma) and 2.5% of foetal calf serum in PBS. Cells were passed through a 70 µm strainer (Falcon) and resuspended in FACS buffer (1% of FCS + 2 mM EDTA in PBS) and used for FACS staining.<br>For cell preparation of whole skin, ears were cut and incubated 1h30 at 37°C with 0.25 mg/ml Liberase TL (Roche), 0.5 mg/ml DNase I in RPMI basic medium. Cells were passed through a 70µm strainer, resuspended in FACS buffer and used for staining.<br>For cell preparation of EDLNs, EDLNs were dissociated with piston, passed through a 70 µm strainer and resuspended in FACS buffer, counted and used for FACS staining.
Instrument	BD Fortessa X20
Software	BD FACS Diva v8<br>FlowJo v10
Cell population abundance	No cell sorting was performed
Gating strategy	For Figures 1 and 3: SSC/FSC was used to exclude debris. Singlets were gated on SSC-W/SSC-H. Alive cells were gated as PI-. Then in the skin, IL-4 and IL-13 was analyzed in CD45+ TCR-b+ cells.<br>In the lymph nodes, Tfh cells were gated as CD4+ PD-1+ CXCR5+. GC B cells were gated as CD19+ B220+ GL7+ CD95+. IgE+ cells were gated as CD19+ B220+ IgE+. IgG1+ cells were gated as CD19+ B220+ IgG1+.<br><br>For Figure 5, SSC/FSC was used to exclude debris. Alive cells were gated as DAPI-. Singlets were gated on SSC-W/SSC-H. Gr-1int cells were gated as CD45+ SiglecF- CD49b- Gr-1int. Gr-1hi cells were gated as CD45+ SiglecF- CD49b- Gr-1hi. TCR-b+ cells were gated as CD45+ SiglecF- TCR-b+. Eosinophils were gated as CD45+ SiglecF+. Basophils were gated as CD45low SiglecF- CD49b+.

- [x] Tick this box to confirm that a figure exemplifying the gating strategy is provided in the Supplementary Information.
